# Coyote use of prairie dog colonies is most frequent in areas used by American badgers

**DOI:** 10.1093/jmammal/gyae066

**Published:** 2024-06-28

**Authors:** Rebecca M Windell, Larissa L Bailey, Travis M Livieri, David A Eads, Dean E Biggins, Stewart W Breck

**Affiliations:** Department of Fish, Wildlife and Conservation Biology, Colorado State University, 1474 Campus Delivery, Fort Collins, CO 80523, United States; Department of Fish, Wildlife and Conservation Biology, Colorado State University, 1474 Campus Delivery, Fort Collins, CO 80523, United States; Prairie Wildlife Research, P.O. Box 643, Stevens Point, WI 54481, United States; U.S. Geological Survey Fort Collins Science Center, 2150 Centre Avenue # C, Fort Collins, CO 80526, United States; U.S. Geological Survey Fort Collins Science Center, 2150 Centre Avenue # C, Fort Collins, CO 80526, United States; USDA National Wildlife Research Center, 4101 Laporte Avenue, Fort Collins, CO 80521, United States

**Keywords:** American Badger, Black-footed Ferret, Coyote, endangered species, intraguild predation, coyote, depredación intragremial, especies en peligro de extinción, hurón de pies negros, tejón americano

## Abstract

The consequences of intraguild predation on vulnerable subordinate species are an important consideration in the recovery of endangered species. In prairie ecosystems, coyotes (*Canis latrans*) are the primary predator of endangered black-footed ferrets (*Mustela nigripes*; hereafter, ferrets) and presumably compete for prairie dog (*Cynomys* spp.) prey. Coyote predation of ferrets is thought to occur at night when ferrets are active aboveground; however, the apparent source of competition, diurnal prairie dogs, are belowground and inaccessible to coyotes at this time, presenting a perplexing temporal mismatch between actual and expected times that coyotes and ferrets come into conflict. Our study used remote wildlife cameras, occupancy models, and overlap of circadian activity patterns to investigate how landscape features, prairie dog colony attributes, and attraction to sympatric species, i.e., American badgers (*Taxidea taxus*; hereafter, badgers) and lagomorphs (cottontail rabbits and jackrabbits) influence Coyote use of prairie dog colonies and potential Coyote–ferret interactions. We first evaluated Coyote use (i.e., occupancy) between prairie dog colonies and surrounding available grasslands, finding that coyotes whose home ranges include prairie dog colonies used colonies nearly twice as much as surrounding grasslands. Next, we investigated biotic and abiotic factors that may influence Coyote use and frequency of use (i.e., detection probability) on prairie dog colonies. We found high Coyote use across all areas on prairie dog colonies; however, their frequency of use increased in areas that were also used by badgers. High overlap between Coyote and badger activity patterns (81%) further supports the spatial use patterns revealed by our occupancy analysis, and badgers and coyotes are known to form hunting associations. Interspecific competition and overlapping patterns of resource use between badgers and ferrets have been documented in previous studies; our study supports these findings and suggests that Coyote attraction to badger activity may influence Coyote–ferret interactions.

## Teaser

Coyotes are the primary predator of endangered black-footed ferrets, a species that selects similar resources and competes with American badgers for prairie dog prey. Our study found that coyotes use prairie dog colonies preferentially to surrounding grasslands, and on colonies, coyotes use areas more frequently that are also used by American badgers. This suggests that Coyote attraction to American Badger activity may influence where and how coyotes and black-footed ferrets interact.

## Abstract (es)

Las consecuencias de la depredación intragremial sobre especies subordinadas vulnerables son una consideración importante en la recuperación de especies en peligro de extinción. En ecosistemas de pradera, los coyotes (*Canis latrans*) son el depredador primario de los hurones de pies negros (*Mustela nigripes*; en adelante, hurones) en peligro de extinción y presumiblemente compiten por las presas de los perritos de las praderas (*Cynomys* spp.). Se cree que la depredación de hurones por parte de los coyotes ocurre durante la noche, cuando los hurones están activos en la superficie; sin embargo, la fuente aparente de competencia, los perritos de las praderas diurnos, están bajo tierra y son inaccesibles para los coyotes en este momento, lo que presenta un desconcertante desajuste temporal entre los momentos reales y esperados en que los coyotes y los hurones entran en conflicto. Nuestro estudio utilizó cámaras trampa, modelos de ocupación y superposición de patrones de actividad circadiana para investigar cómo las características del paisaje, los atributos de las colonias de perritos de las praderas y la atracción por especies simpátricas (es decir, tejones americanos, *Taxidae taxus* y lagomorfos) influyen en el uso de las colonias de los perritos de las praderas por parte de los coyotes e interacciones potenciales entre coyote-hurón. Primero evaluamos el uso de coyotes (es decir, ocupación) entre las colonias de perritos de las praderas y los pastizales circundantes disponibles, y encontramos que los coyotes cuyo territorio colinda con colonias de perritos de la pradera usaban las colonias casi el doble ($\hat{\Psi }\;$ = 0,77, IC del 95% [0,48, 0,93]) que los pastizales circundantes ($\hat{\Psi }\;$ = 0,37; IC del 95% [0,21; 0,57]). Después investigamos los factores bióticos y abióticos que pueden influir en el uso y la frecuencia de uso de los coyotes (es decir, la probabilidad de detección) en las colonias de perritos de las praderas. Encontramos un alto uso por parte de los coyotes en todas las áreas de las colonias de perritos de las praderas; sin embargo, su frecuencia de uso aumentó en áreas que también eran utilizadas por tejones (β = 3,95, IC del 95% [2,35, 5,58]). La alta superposición entre los patrones de actividad de coyotes y tejones (81%) respalda aún más los patrones de uso espacial revelados por nuestro análisis de ocupación, y se sabe que los tejones y los coyotes forman asociaciones de caza. En estudios previos se han documentado la competencia interespecífica y los patrones superpuestos de uso de recursos entre tejones y hurones; nuestro estudio respalda estos hallazgos y sugiere que la atracción del coyote por la actividad del tejón puede influir en las interacciones coyote-hurón.

Intraguild predation is an important form of competition where a dominant predator benefits from killing—and sometimes eating—a subordinate predator, resulting in reduced competition for shared prey and other resources (Polis et al. 1989; Polis and Holt 1992; Palomares and Caro 1999). Mortality from this type of interaction is an important consideration in many systems and is particularly relevant in the recovery of endangered species (Connell and Slatyer 1977; Goodrich and Buskirk 1995). In prairie ecosystems, coyotes (*Canis latrans*; Bekoff 1977) and endangered black-footed ferrets (*Mustela nigripes*; hereafter, ferrets; Hillman and Clark 1980) both prey on prairie dogs (*Cynomys* spp.; Biggins 2000; Chronert 2007) and coyotes appear to be the primary predator of ferrets, accounting for up to 67% of predation-based mortalities (Breck et al. 2006; Eads et al. 2015). However, this apparent example of intraguild predation is perplexing based on 2 primary observations.

First, intraguild predation is thought to occur most frequently between more taxonomically related species, those with high dietary overlap, and between species with intermediate levels of body size overlap (i.e., when the dominant predator is 2 to 5.4 times larger than the subordinate predator; Donadio and Buskirk 2017). In contrast, coyotes and ferrets are members of different taxonomic families, only have moderate dietary overlap (i.e., prairie dogs comprise ~60% to 90% and ~38% of ferret and Coyote diets, respectively; Campbell et al. 1987; Chronert 2007; Brickner et al. 2014), and coyotes are approximately 13 times larger than ferrets (Bekoff 1977; Biggins and Schroeder 1987). Second, ferrets are highly specialized predators of prairie dogs and have evolved behaviors and fossorial morphology that allow them to hunt prairie dogs belowground at night and at burrow openings in the morning (Eads et al. 2010). Coyotes lack these fossorial adaptations and are commonly observed employing a “sit-and-wait” hunting strategy at prairie dog burrow openings during daytime and crepuscular periods (e.g., Lehner 1981; Slobodchikoff 2002; Kiriazis and Slobodchikoff 2006). Moreover, Coyote predation on ferrets occurs mostly at night (Biggins 2000; Breck et al. 2006) when prairie dogs are underground and presumably inaccessible to coyotes. Thus, it is unclear what factor(s) attract coyotes to prairie dog colonies at night and influence Coyote–ferret interactions.

We addressed questions of Coyote use of prairie dog colonies by investigating preferences and predictors that may provide insight into primary influences on Coyote–ferret interactions. We did this by considering a variety of hypotheses about Coyote use of prairie dog colonies including the influence of landscape features, prairie dog colony attributes, alternative prey sources, intraguild interactions, and community activity patterns (Table 1). Although coyotes have been observed at higher densities and in greater relative abundance on prairie dog colonies in comparison to uncolonized grasslands (Krueger 1986; Ceballos et al. 1999), it remains to be determined if coyotes use prairie dog colonies differently in relation to surrounding grasslands (Chronert 2007). In addition, there is considerable uncertainty about the strength of various abiotic, biotic, and temporal factors that could predict Coyote use of prairie dog colonies (e.g., Biggins 2000; Poessel et al. 2011; Eads et al. 2015).

**Table 1.** Variables used in 2 occupancy analyses to evaluate Coyote habitat preferences and predictors of Coyote use in Badlands National Park and Buffalo Gap National Grasslands, South Dakota in 2018. In the first analysis, we modeled Coyote probability of use of camera units located on prairie dog colonies and in surrounding grassland habitats (Model Set 1). The second analysis used only camera units on prairie dog colonies and investigated the influence of landscape features, prairie dog colony attributes, and interspecific attractions on Coyote use (Model Set 2). Variables were used to model both Coyote use (i.e., occupancy; Ψ) and frequency of use (i.e., detection probability; *p*) for both analyses.

Variable	Model set	Parameters	Range	Description
Habitat	1	Ψ	Binary	Variation between prairie dog colony and grassland habitat types
Landscape features				
Rip	2	Ψ, *p*	1 to 1,094 m	Distance of camera to nearest riparian area
Stream	2	Ψ, *p*	31 to 705 m	Distance of camera to nearest stream or gully
Two-Track	2	Ψ, *p*	0 to 3,225 m	Distance of camera from nearest two-track road
Colony attributes				
Edge	2	Ψ, *p*	0 to 930 m	Distance of camera from edge of colony
Size	2	Ψ, *p*	131 to 1,519 m	Size of prairie dog colony for a given camera
PD Coarse	2	Ψ, *p*	0 to 54 pd^a^/ha	Prairie dog density measured coarsely across a 4-ha area encompassing each camera
PD Fine	2	Ψ, *p*	1 to 52 pd/ha	Prairie dog density measured finely within a 30-m buffer of camera
Interspecific attractions				
Badger	2	Ψ, *p*	0.15 to 1^b^	Conditional probability of badger use
Badger Fine	2	*p*	Binary	Whether or not a badger was detected in a survey at a given camera
Nuisance variables				
Rotation	1	Ψ	Binary	If a camera was included in the first (June) or second (July) rotation of cameras
Veg	1	*p*	1.5 to 8.9^c^	Average vegetative visual obstruction at a camera
Brown	1, 2	*p*	Binary	Use of Browning camera
Bush	1	*p*	Binary	Use of Bushnell camera
Effort	1	*p*	0 to 1	The proportion of days within a survey that a given camera was functional

^a^pd = prairie dogs.

^b^Conditional probability of badger occupancy.

^c^Visual obstruction via Robel pole method.

Landscape features have been documented as important predictors of predation risk in carnivore systems (Hebblewhite et al. 2005; Kauffman et al. 2007) and preferential use of certain features by coyotes may increase ferret predation risk in proximity to these areas. Poessel et al. (2011) modeled Coyote use of prairie dog colonies in relationship to linear features and movement corridors (i.e., roads, drainages, fence lines) in an attempt to predict Coyote predation risk on ferrets and did not find evidence that these features increased risk; however, other landscape features—such as those that provide vegetative structures (e.g., for sit-and-wait hunting; Jung 2021) across portions of otherwise open prairie habitats—have yet to be explored and may be important predictors of Coyote use of prairie dog colonies.

Attributes such as prairie dog density, colony size, or location within a colony could also influence how coyotes use prairie dog colonies (Eads et al. 2015). If coyotes and ferrets are both attracted to areas on colonies with similar densities of prairie dogs, this could indicate direct competition for prairie dogs and suggest that Coyote predation on ferrets may result from chance encounters in these areas despite an apparent temporal mismatch in when each species would be most likely to hunt for prairie dog prey (e.g., Slobodchikoff 2002; Kiriazis and Slobodchikoff 2006). However, an important consideration in assessing attraction to particular prairie dog densities may depend on the scale at which coyotes select areas to use on colonies. For example, fine-scale prairie dog densities likely reflect the social structure of prairie dog family groups that form across colonies, which create higher density pockets that are preferentially used by both American badgers (*Taxidea taxus*; hereafter, badgers; Long 1973) and ferrets (Biggins et al. 1993, 2006; Eads et al. 2011, 2013; Grassel and Rachlow 2018). In contrast, coyotes primarily rely on visual cues and focus their attention over wider areas while hunting (Horn and Lehner 1975; Wells 1978; Minta et al. 1992), and as a result prairie dog densities across broad swaths of colony may be a more relevant scale for understanding Coyote use of prairie dog colonies.

In addition to prairie dog prey biomass, colonies are also rich sources of alternative prey (Agnew et al. 1986; Krueger 1986) and coyotes may be attracted to areas used by other prey species. Small mammal prey, including cottontail rabbits (*Sylvilagus* spp.), are a ubiquitous component of Coyote diets and rabbit densities have been reported as being up to 27 times higher on prairie dog colonies in comparison to surrounding grasslands (Hansen and Gold 1997). Further, the activity patterns of both coyotes and lagomorphs—cottontail rabbits and jackrabbits (*Lepus* spp.)—overlap during nocturnal and crepuscular periods (Mech et al. 1966; Arias-Del Razo et al. 2011) and thus nocturnal rabbit activity may facilitate opportunistic Coyote intraguild predation of ferrets. For example, Eads et al. (2015) found that coyotes selected for areas of prairie dog colonies used by rabbits, although rabbits did not appear to select areas of colonies with high prairie dog densities.

Coyote–ferret interactions may also be shaped by other members of the carnivore guild such as badgers. Badgers have strong, specialized thoracic digging limbs, prefer high-density portions of prairie dog colonies, and selectively excavate burrows in areas used by ferrets, presumably to unearth prairie dogs that ferrets have killed and potentially cached (Eads et al. 2013, 2016). While badgers exert intraguild predation pressures on ferrets and will consume the ferrets that they kill, badgers only account for approximately 5% of all predation-based ferret mortalities (Biggins 2000). Coyotes, however, can be attracted to badgers actively excavating burrows (Eads et al. 2016) and have been observed hunting in association with badgers (Minta et al. 1992). In prairie ecosystems where coyotes and badgers are both hunting for semifossorial prey such as prairie dogs and other ground squirrels, the hunting associations formed between these 2 predators appear to be mutually beneficial. In its simplest form, the badger attempts to catch belowground prey by excavating burrows and the Coyote waits aboveground to snatch animals attempting to escape through interconnected burrow openings. It is believed that the sentinel presence of the Coyote aboveground benefits the badger by keeping prey belowground, and the Coyote benefits by catching animals fleeing from belowground pursuit by the badger (Minta et al. 1992).

Our primary objective was to investigate multiple hypotheses for Coyote use of prairie dog colonies (Table 1) and better understand spatial and temporal influences of Coyote use to inform ferret conservation. Using wildlife cameras and an occupancy approach, we first determined if coyotes preferentially use prairie dog colony habitats compared to surrounding grasslands (Hall et al. 1997). Second, we investigated predictors of Coyote use and frequency of use on prairie dog colonies, including landscape features (e.g., travel routes, riparian areas), prairie dog colony attributes (e.g., prairie dog density, colony size, proximity to colony edge), use by alternative prey (i.e., lagomorphs), and interspecific interactions with—or attractions to—other members of the predator community (i.e., badgers). Third, we investigated the circadian patterns of coyotes on prairie dog colonies in comparison to adjacent grasslands and nocturnal periods in which ferrets are known to be most active, including the late hours of the night (0100 to 0300 h; Biggins 2000). Finally, we assessed activity overlap of coyotes with prairie dogs, lagomorphs, and badgers to draw connections between spatial and temporal influences of Coyote attraction to prairie dog colonies.

## Materials and methods

### Study area

**Fig. 1.**
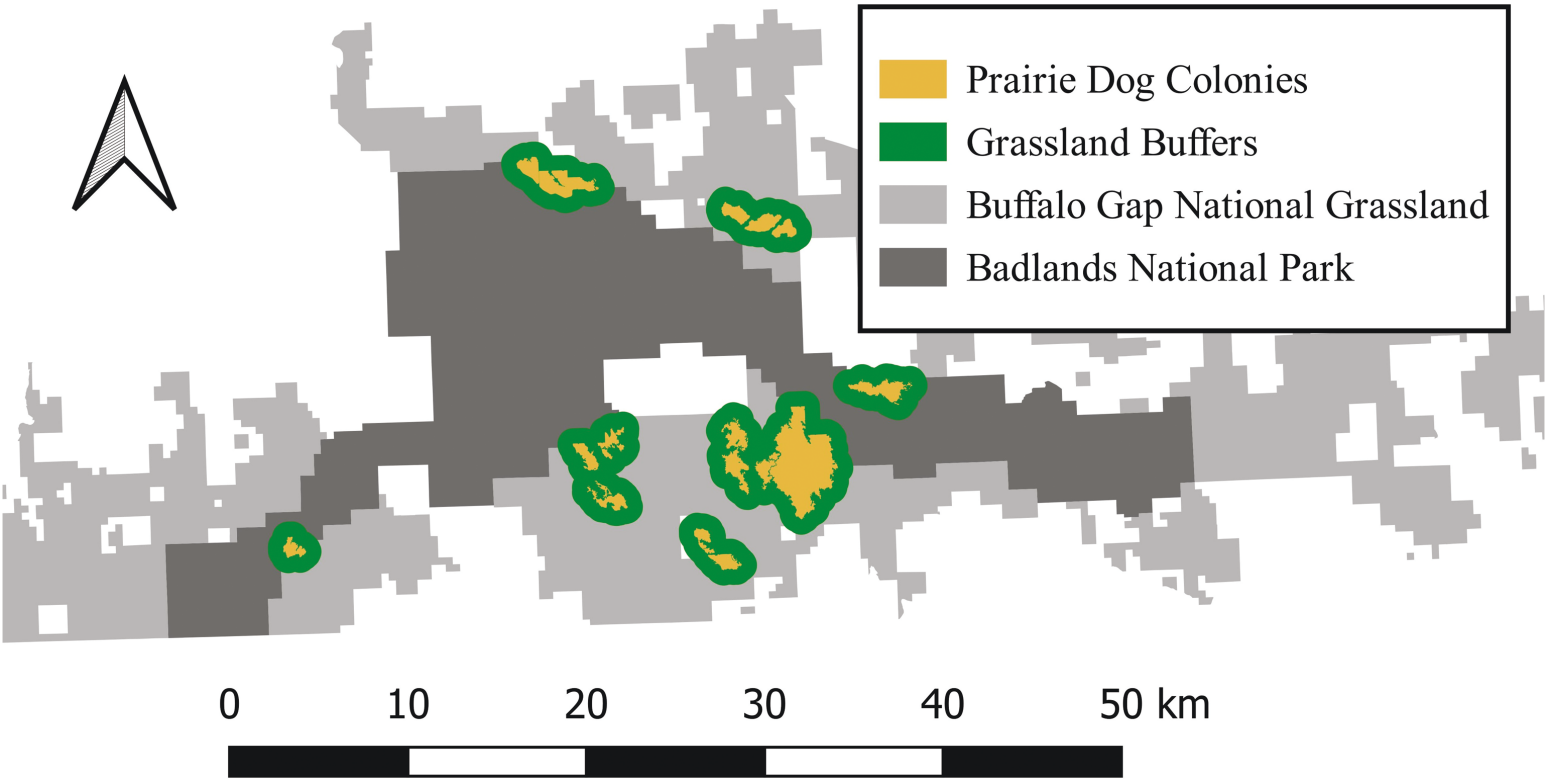
Large (>65 ha) prairie dog colonies with known Black-footed Ferret occurence in Badlands National Park and Buffalo Gap National Grasslands, South Dakota, were sampled using wildlife camera traps (*n* = 118) in an occupancy framework in 2018. Prairie dog colonies and an 850-m buffer of grassland habitat surrounding colonies were sampled to evaluate Coyote habitat preferences and assess the strength of predictors of Coyote use on prairie dog colonies in the context of Black-footed Ferret conservation.

Our study was conducted between 24 June and 30 August 2018 in the northern unit of Badlands National Park (BNP) and the surrounding Buffalo Gap National Grasslands (BGNG) in southwestern South Dakota (Fig. 1). Together, BNP and BGNG contain 5,875 ha of occupied Black-tailed Prairie Dog (*Cynomys ludovicianus*; Hoogland 1996) colonies and are home to the largest population of wild black-footed ferrets (~115 individuals in 2018). BNP and BGNG were dominated by mixed-grass prairie where prairie dog colonies were denoted by the presence of close-cropped grasses, burrow mounds, and the prevalence of small forbs and shrubs (Agnew et al. 1986; Livieri and Anderson 2012). Primary BGNG land uses included hunting and the grazing of both Domestic Cattle (*Bos taurus*) and Bison (*Bison bison*; Meagher 1986). Primary BNP land uses were recreation, tourism, and sanctuary for species such as Bison, Bighorn Sheep (*Ovis canadensis*; Shackleton 1985), and Pronghorn Antelope (*Antilocarpa americana*; O’Gara 1978). Topography of the area was primarily flat with interspersed arroyos, bands of layered Badlands rock formations, and riparian areas that contained Eastern Cottonwood trees (*Populus deltoides*). Two state highways (240 and 44) and 2 county highways (509 and 590) interconnected BNP and BGNG with an assortment of minimally traveled forest service roads and fence lines throughout the area. Although BNP and BGNG were managed differently, the 2 areas formed 1 contiguous study area (Fig. 1).

Personnel from BNP and BGNG conducted biannual Black-tailed Prairie Dog surveys where colonies were mapped using GPS units and prairie dog density was roughly estimated as high, medium, or low. Using data from 2017 surveys, we identified the largest, intact prairie dog colonies (greater than 65 ha) with known ferret presence. Where multiple prairie dog colonies greater than 65 ha occurred in close proximity, we clustered colonies into subcomplexes of interconnected habitat (Biggins et al. 2006). Using these criteria, we identified 8 prairie dog colony subcomplexes (hereafter referred to as colonies) that were included in our study—6 in BGNG and 2 in BNP (Fig. 1). We delineated an 850-m buffer around each colony, where our buffer distance was determined by the radius of the average Coyote core range in our study area (1.5 km^2^; Schroeder 2007). These buffers comprised grasslands available to coyotes that used or had access to prairie dog colonies and were delineated to compare Coyote use between prairie dog colonies and surrounding grasslands. Here, it is important to note that these grassland buffers would only partially capture the core ranges of coyotes with territories that comprised primarily of grassland habitat.

### Analytical approach

We used camera surveys to investigate: (1) if Coyote use was higher on prairie dog colonies compared to adjacent grasslands; (2) what biotic and abiotic predictors helped explain patterns of Coyote use on prairie dog colonies; and (3) if Coyote activity varied between prairie dog colonies and grasslands. The first 2 objectives involved conducting 2 separate occupancy analyses with the first incorporating data from both prairie dog colonies and surrounding grasslands, and the second only using data from prairie dog colonies. We used results from the first analysis to limit the number of “nuisance” variables (see Nuisance variables below) that we included in the second analysis. For the third objective we conducted an activity analysis using counts of camera detections.

### Camera surveys

We placed remote wildlife cameras in our 2 habitats of interest (i.e., prairie dog colonies and surrounding grasslands) and collected data during 2 30-day rotations, the first from 24 June to 24 July 2018 and second from 31 July to 30 August 2018. We randomly placed 4 cameras at least 400 m apart in both habitats at each of the 8 colonies, resulting in a total of 32 cameras on prairie dog colonies and 32 cameras on grasslands during each of our 2 rotations (total camera locations = 128). During the first rotation, 1 BGNG colony was inaccessible due to muddy conditions and these cameras were redistributed to the 2 BNP colonies, resulting in 6 cameras on colonies and grasslands at these locations. Due to camera malfunctions and heavy cattle/bison activity, we were unable to gather data at 10 of our camera sites, resulting in data from a total of 61 camera locations on prairie dog colonies and 57 camera locations on surrounding grasslands (total camera locations = 118).

Cameras were placed on t-posts approximately 50 cm from ground level. To increase the probability of Coyote detection on prairie dog colonies, all cameras were placed 7.5 m from the center of the nearest active prairie dog burrow entrance. Cameras on colonies were pointed at the active prairie dog burrow entrance at a randomly selected cardinal azimuth. Active burrows were determined by the presence of fresh scat (i.e., greenish, black, or dark brown), visual observation of prairie dogs, and mound scratching activity (Biggins et al. 1993). Cameras on grasslands were placed 7.5 m from the nearest feature that would increase Coyote detection (i.e., 2-track road, game trail, cattle trail, drainage entrance). Based on an average camera detection range of 30 m and a 45-degree field of view (Trailcampro.com n.d.), the effective area sampled by each camera did not extend past this 30-m radius. Thus, we define each sample unit as the ~0.28-ha area around each camera, with camera placement designed to ensure independence among resulting camera detection histories (MacKenzie et al. 2017).

Three different brands of camera traps were used in our study. Brands and their respective models included Browning BTC Series (*n* = 30), Bushnell Trophy Series (*n* = 23), and Cuddeback Longrange IR (*n* = 11). Cameras were systematically placed across each colony to ensure interspersed distribution of camera brands. We programed all cameras similarly with a medium sensitivity, the smallest delay possible between photographs (≤1 s), and in bursts of 3 photos. All cameras were equipped with infrared flash to reduce the effects of flash on Coyote behavior.

### Camera data

Camera data were processed using the Colorado Parks and Wildlife Photo Warehouse software (Ivan and Newkirk 2016), where each photo was identified to species by 2 independent observers (including the lead author), except in cases of very large photo sets (i.e., at camera units that had >10,000 photos due to grass blowing in the wind or high cattle/bison activity) where only 1 observer identified species in the photo set. The lead author verified species for any images that were not congruently identified by 2 observers, and all species in large photo sets. Camera data were used to construct detection histories for each camera unit for occupancy modeling; specifically, we divided the 30 days of sampling into 10 sampling occasions (i.e., each survey consisted of 3 days). We considered detection events to be independent if detections were >1 h apart (Lewis et al. 2015; Lendrum et al. 2017), censored data from any periods in which cameras malfunctioned for the entirety of a survey (e.g., dead batteries, camera knocked down by cattle), and accounted for malfunctions during only a portion of each survey (see Nuisance variables below).

### Landscape features

We identified 3 landscape variables that could influence Coyote use at camera units in our study area: distance to riparian areas (*Rip*); distance to 2-track roads (*Two-Track*); and distance to streams and gullies (*Stream*; Table 1). Riparian areas were point locations that contained larger bodies of water such as ponds and livestock reservoirs, and that were surrounded by vegetation and topography that could provide cover, water, and den sites across the otherwise open and relatively unprotected grasslands. Two-track BGNG roads and service roads within BNP could serve as travel corridors for coyotes. Streams and gullies could serve multiple purposes including areas of cover, water sources, den sites, and travel corridors, with the largest distinction between these areas and riparian areas being that they were continuous linear features. We predicted that as distance to each of these 3 variables decreased, Coyote use would increase. Shapefiles of riparian areas, 2-track roads, streams, and gullies were acquired from the USDA Data Gateway and we used ArcMap (Esri, Redlands, California) to measure the distance (m) to each of these features from our camera locations.

### Prairie dog colony attributes

We considered 4 attributes to investigate the role of prairie dogs in Coyote use on colonies: fine-scale prairie dog density at each camera unit (*PD Fine*); broad-scale prairie dog density measured across each colony (*PD Coarse*); colony size (*Size*); and distance from each camera to the colony edge (*Edge*; Table 1). Because the scale at which coyotes select for prairie dog density is unknown, we measured prairie dog densities at fine and coarse scales, in addition to calculating the total amount of prairie dog colony available (i.e., *Size*). If Coyote use varied in relationship to distance from edge of the colony (i.e., *Edge*), this metric could provide information about patterns of preference with proximity to adjacent grasslands or preferences related to patterns of succession on prairie dog colonies. For example, colony centers are typically the oldest, most established portions of colonies and feature the most altered vegetative structure in comparison to surrounding grasslands (Krueger 1986). To measure fine-scale prairie dog density (*PD Fine*), we counted all burrows within a 30-m buffer of each camera (0.28 ha) and classified each burrow as active or inactive. Burrows were considered active based on the same criteria used to select active burrows for camera placement (Biggins et al. 1993). We used the linear relationship Biggins et al. (1993) reported between Black-tailed Prairie Dog active burrow counts and individual animals to estimate prairie dog abundance (0.179 × number of active burrows). Fine-scale density per hectare was calculated by dividing estimated prairie dog abundance by the number of hectares sampled. To measure broad-scale prairie dog density (*PD Coarse*), we used a modification of the strip transect technique of Biggins et al. (1993). The modified technique consists of counting the number of active and inactive prairie dog burrows along strip transects spaced 200 m apart and running the entire length of the colony using an all-terrain vehicle outfitted with a 3-m stretch of electrical conduit pipe. Strip transects sampled approximately 1.5% of 4-ha plots delineated across the entirety of each focal colony (Biggins et al. 1993). In ArcGIS we extracted colony size and the distance of each camera location to the edge of prairie dog colonies from BGNG and BNP prairie dog colony surveys.

### Interspecific attractions

We were interested in Coyote attraction to badgers, ferrets, and lagomorphs (i.e., *Sylvilagus* spp., *Lepus townsendii*). Due to very few ferret and lagomorph detections on prairie dog colonies, we were unable to estimate conditional occupancy probabilities or calculate activity overlap for these species. This was expected for ferrets due to their limited numbers; however, for lagomorphs this was unexpected and may represent a low period in the lagomorph population cycle or the effect of a 2017 drought. For badgers, we calculated unit-specific conditional occupancy estimates (Ψ_conditional_) based on the null (or constant) structure for probability of use and the best supported detection structure including nuisance variables (see Nuisance variables below; Supplementary Data SD1). Conditional occupancy probabilities are 1 for all camera units where badgers were detected and provided an estimate of occupancy, conditional on survey results, for all other units. We included this unit-specific covariate to assess whether badger use (*Badger*) influenced Coyote use or frequency of use (Table 1; MacKenzie et al. 2017). To evaluate whether the detection of badgers during a given 3-day survey influenced Coyote detection during that same survey, we included detections of badgers (*Badger Fine*) as survey-specific covariates when modeling Coyote detection probability on prairie dog colonies (Massara et al. 2016); Table 1).

### Nuisance variables

Camera brand was identified as a key predictor of variation in detection probability in a concurrent study that used the same 3 camera brands (Windell et al. 2022); thus, we included camera brand as a covariate in our first Coyote occupancy analysis (Table 1). Browning cameras (*Brown*) increased Coyote detection probability in comparison to Bushnell (*Bush*) and Cuddeback cameras; thus, we used Browning cameras as a covariate in our second occupancy analysis (Table 1). Camera brands were distributed as evenly as possible across colonies to avoid any bias associated with their placement. Cameras were sometimes ripped from t-posts due to rubbing activities from cattle and bison and thus not operational during the entire survey. As a result, we developed a covariate that reflected the proportion of each survey that a camera was functional (*Effort*) and included this covariate in our first analysis.

In addition to camera related nuisance variables, we accounted for the potential influence of: (1) camera rotation (*Rotation*) on Coyote use of units to account for any variation between our 2 sampling periods; (2) the effects of each 3-day survey period (*Survey*) on detection probability; and (3) vegetation height (*Veg*) on detection probability. Vegetation height was measured at the time of camera setup using the Robel pole method to determine visual obstruction (i.e., VO) at each camera unit (Robel et al. 1970). Given an approximate camera field of view of 45 degrees (Trailcamerapro.com 2023), we identified two 30-m transects at the 15-degree and 30-degree marks within the field of view. Two VO measurements were recorded on opposite sides of each transect line at 2-m intervals, for a total of 60 measurements per camera unit. Measurements from the 2 transects were then averaged to provide a single VO value for each camera unit (*Veg*).

### Occupancy analyses

For wide-ranging species like coyotes, occupancy (Ψ) estimates are interpreted as the probability that a unit is “used” rather than “occupied” by the species of interest during the specified season (i.e., the 30-day period; MacKenzie et al. 2017). In addition, detection probability, or the probability that the species is detected during a survey at a used unit, is related to the relative use of a unit or local species abundance (Royle et al. 2003; Mackenzie and Royle 2005; Lewis et al. 2015). When modeled with covariates, detection probability can provide important information about the frequency that a unit was used (i.e., fine-scale measurement of use during a 3-day survey at used units).

We conducted 2 separate occupancy analyses using single-season models to address our objectives. In the first analysis, we used all 118 camera units to investigate Coyote use preferences between prairie dog colony and grassland habitat (Table 1). Specifically, we: (1) tested whether Coyote probability of use was higher for camera units located on prairie dog colonies relative to those in the surrounding grassland habitat; and (2) explored the importance of nuisance variables on Coyote use and frequency of use (i.e., detection probability). This first analysis considered *Veg*, *Brown*, *Bush*, and *Effort* on detection probability (see Nuisance variables above) and the effect of *Habitat* (i.e., prairie dog colony or grasslands) and *Rotation* on Coyote use (Tables 1 and 2).

**Table 2.** Model selection results for Coyote use (Ψ) and detection probability (*p*, frequency of use) for camera units in 2 different habitat types (*Habitat*), prairie dog colonies and surrounding grasslands, in Badlands National Park and Buffalo Gap National Grassland, South Dakota in 2018. Probability of Coyote use was modeled as a function of seasonal effects between camera rotations (*Rotation*), *Habitat*, and additive (+) or interactive (*) effects of these covariates. Detection probability structures include the potential influence of camera type (Browning = *Brown*; Bushnell = *Bush*), proportion of each survey that a camera was operational (*Effort*), and visual obstruction from vegetation (*Veg*). The null model, where Coyote use and frequency of use are constant across all units and surveys, Ψ(.), *p*(.), is given as a reference. Model selection statistics include: AIC_c_ = Akaike’s Information Criterion adjusted for small sample bias; *w*_*i*_ = AIC_c_ model weights; *K* = number of model parameters and a measure of model fit (deviance = −2log(likelihood)).

Model^a^	AIC_c_	ΔAIC_c_	*w*_*i*_	*K*	Deviance
Ψ(Habitat), *p*(Brown)	485.16	0	0.458	4	476.81
Ψ(Rotation + Habitat), *p*(Brown)^a^	486.48	1.32	0.236	5	475.95
Ψ(Rotation * Habitat), *p*(Brown)	487.75	2.59	0.125	6	475.00
Ψ(Rotation * Habitat), *p*(Brown + Veg)	489.33	4.17	0.057	7	474.31
Ψ(Rotation * Habitat), *p*(Brown + Bush)	489.49	4.33	0.053	7	474.47
Ψ(Rotation * Habitat), *p*(Brown + Bush + Effort)	491.32	6.15	0.021	8	474.00
Ψ(.), *p*(Brown)	491.49	6.32	0.019	3	485.28
Ψ(Rotation), *p*(Brown)	491.82	6.66	0.016	4	483.47
Ψ(Rotation * Habitat), *p*(Brown + Bush + Effort + Veg)	493.03	7.86	0.009	9	473.36
Ψ(Rotation * Habitat), *p*(Bush)	493.48	8.32	0.007	6	480.72
Ψ(Rotation * Habitat), *p*(Veg)	495.33	10.16	0.003	6	482.57
Ψ(Rotation * Habitat), *p*(Effort)	495.67	10.50	0.002	6	482.91
Ψ(.), *p*(.)	496.58	11.42	0.002	2	492.48

^a^Model structures with uninformative or pretending variables (Arnold 2010).

Retaining the influential nuisance variables, the second analysis conditioned on camera units on prairie dog colonies (*n* = 61 camera units; Table 1) to determine factors influencing Coyote use and frequency of use on prairie dog colonies. Here, we investigated patterns of Coyote use and frequency of use in relationship to landscape features, prairie dog colony attributes, and interspecific attractions using a stepwise approach. Given the large number of variables of interest, we modeled effects on Coyote occupancy (i.e., use) as the first step in this analysis and then carried the most supported occupancy structure into the second step, where we modeled effects on Coyote detection probability (i.e., frequency of use; Supplementary Data SD2). Finally, we combined all supported occupancy structures from the first step with the most supported detection probability structure from the second set to create a final model set (Table 3).

**Table 3.** Model selection results for the final model set representing the combination supported structures of Coyote use (Ψ) and detection probability (*p*, frequency of use) based on our stepwise modeling approach (Supplementary Data SD2). Models were fit to detection–nondetection data from camera units on 8 prairie dog colonies in Badlands National Park and Buffalo Gap National Grassland, South Dakota in 2018. Factors influencing Coyote use included unit-specific estimates of American Badger use (*Badger*) and both fine- (*PD Fine*) and coarse-scale (*PD Coarse*) prairie dog density. Detection probability structures include the influence of camera type (Browning = *Brown*) and *Badger*. The asterisk sign (*) denotes an interaction effect between covariates, the plus sign (+) denotes an additive effect between covariates, and dot (.) denotes no covariate effect on Ψ or *p*. Model selection statistics include: AIC_c_ = Akaike’s Information Criterion adjusted for small sample bias*; *w*_*i*_ = AIC_c_ model weights; *K* = number of model parameters; deviance = −2log(likelihood), a measure of model fit.

Model^a^	AIC_c_	ΔAIC_c_	*w*_*i*_	*K*	Deviance
Ψ(.), *p*(Brown + Badger)	309.12	0.00	0.47	4	300.41
Ψ(PD Coarse), *p*(Brown + Badger)^a^	311.36	2.24	0.15	5	300.27
Ψ(Badger), *p*(Brown + Badger)^a^	311.38	2.26	0.15	5	300.29
Ψ(PD Fine), *p*(Brown + Badger)^a^	311.47	2.35	0.15	5	300.38
Ψ(Badger + PD Coarse), *p*(Brown + Badger)^a^	313.48	4.36	0.05	6	299.93
Ψ(Badger * PD Coarse), *p*(Brown + Badger)^a^	315.62	6.50	0.02	7	299.51
Ψ(Badger), *p*(Brown)	322.97	13.85	0.00	4	314.26
Ψ(Badger + PD Coarse), *p*(Brown)	324.15	15.03	0.00	5	313.06
Ψ(Badger * PD Coarse), *p*(Brown)	326.03	16.91	0.00	6	312.47
Ψ(PD Fine), *p*(Brown)	326.12	17.00	0.00	4	317.41
Ψ(PD Coarse), *p*(Brown)	326.43	17.31	0.00	4	317.71
Ψ(.), *p*(Brown)	326.84	17.72	0.00	3	320.42

^a^Model structures with uninformative or pretending variables (Arnold 2010).

All models were constructed and fit in Program MARK (White and Burnham 1999) and we used AIC_c_ selection to assess model performance (Burnham and Anderson 2002). We also ran bootstrap Chi-squared goodness-of-fit tests using the most parameterized model in each analysis and estimated overdispersion ($\hat{c}$) using 10,000 simulations in Program PRESENCE (MacKenzie and Bailey 2004; Hines 2006). Prior to modeling, we tested for correlations among measured covariates using Pearson’s product-moment correlation and found that none of the covariates correlated above the maximum value of |*r*| > 0.7 (Dormann et al. 2013).

### Circadian activity

To understand if Coyote temporal activity varied between prairie dog colonies and grasslands, we divided each day into 5 distinct periods: Dawn; Day; Dusk; Early Night; and Late Night. We used the *suncalc* package (Agafonkin and Thieurmel 2017) in R (R Core Team 2019) to extract the times of nautical dawn, sunrise end, nautical dusk, sunset start and nadir (i.e., the darkest time of night) to delineate these periods. Day was bound by sunrise end and sunset start, Dusk by sunset start and nautical dusk, Early Night by nautical dusk and nadir, Late Night by nadir and nautical dawn, and Dawn by nautical dawn and sunrise end. Using these categories, we calculated the expected proportion of activity within each time period assuming even activity across all categories (Lendrum et al. 2017). We determined categories at the midpoint of each rotation (i.e., 15 days into each sampling period) and averaged proportions across our 2 rotations, yielding an expected percentage of time in each category as 5.43% for Dawn and Dusk, 60.46% for Day, and 14.33% for Early and Late Night. We used a Chi-squared goodness-of-fit test (α = 0.05) to assess whether the observed proportions of time in each category differed from expected proportions on grasslands and prairie dog colonies. To provide an ecological interpretation of temporal variation in Coyote activity across colonies and between habitat types (i.e., prairie dog colonies and surrounding grasslands), we calculated the differences in proportions of activity in each category as odds ratios. To do this we first transformed the proportion of photos (p) into odds (p/(1 − p)) on prairie dog colonies (p_colonies_) and grasslands (p_grasslands_) and then calculated the ratio of these odds (odds(p_colonies_)/odds(p_grasslands_); Lendrum et al. 2017).

We used the *overlap* package (Meredith and Ridout 2014) in R to determine diel activity patterns for coyotes on prairie dog colonies and grasslands, and between coyotes, badgers, and prairie dogs. We calculated the overlap of activity between these species using a bandwidth adjustment of 0.8 as recommended by Ridout and Linkie (2009) and calculated 95% confidence intervals (CIs) for overlap estimates using 10,000 bootstrap simulations.

## Results

### Camera data

Cameras recorded a total of 233,875 photos over 7,680 trap-nights. Species captured included Pronghorn Antelope (*n* = 6,179), American Badger (*n* = 260), Bighorn Sheep (*n* = 89), Bison (*n* = 57,693), Bobcat (*Lynx rufus*; *n* = 5), Domestic Cattle (*n* = 115,587), Coyote (*n* = 663), Mule Deer (*Odocoileus hemionus*; *n* = 1,777), Porcupine (*Erethizon dorsatum*; *n* = 30), Black-tailed Prairie dogs (*n* = 46,787), cottontail rabbits (*n* = 137), raccoons (*Procyon lotor*; *n* = 12), White-tailed Deer (*Odocoileus virginianus*; *n* = 3,247), and white-tailed jackrabbits (*L. townsendii*; *n* = 11). We recorded 66 and 22 independent detections of coyotes on prairie dog colonies and grasslands, respectively. For the other focal species of interest, we recorded 17, 1, and 820 independent detections of badgers, lagomorphs, and prairie dogs, respectively on prairie dog colonies. Although ferrets were observed during annual population monitoring spotlight surveys in our study area and on camera traps associated with a concurrent study (Windell et al. 2022), we did not detect any ferrets on cameras during the designated time periods used for this study.

### Coyote use on prairie dog colonies versus surrounding grasslands

In our first analysis, the top model held 45.8% of the AIC_c_ weight and indicated that Coyote use was greater on prairie dog colonies ($\hat{\Psi }$ = 0.77, 95% CI [0.48, 0.93]) compared to surrounding grasslands habitats ($\hat{\Psi }$ = 0.37, 95% CI [0.21, 0.57]; Table 2). We found little evidence that Coyote use in a given habitat type varied between our 2 time periods; the inclusion of the *Rotation* covariate showed little improvement in model fit (deviance < 1; Table 2) and the estimated effect was small and imprecise ($\hat{\beta }$ = −0.58, 95% CI [−1.79, 0.64]). Browning cameras had a higher probability of detection ($\hat{p}$ = 0.18, 95% CI [0.14, 0.24]) relative to the other camera types ($\hat{p}$ = 0.07, 95% CI [0.04, 0.12]). There was little support that *Veg*, *Survey*, or *Effort* influenced detection probabilities (Table 2); thus, we did not include these in our analysis focused on Coyote use and frequency of use of prairie dog colonies. Our goodness-of-fit test using the most parameterized model structure ( Ψ (Rotation * Habitat), *p*(Brown + Bush + Effort + Veg)) initially indicated a lack of fit and substantial overdispersion ($\hat{c}$ = 2.97; χ^2^ = 9167; *P* = 0.04); however, this test does not have a way of pooling observations with low expected values (MacKenzie and Bailey 2004) which is nearly inevitable with missing survey data. Two encounter histories accounted for the majority of the test statistic (χ^2^ = 7888) and when these 2 histories were removed, model fit using the remaining 116 histories was adequate (χ^2^ = 2233; *P* = 0.30) and there was no evidence of overdispersion ($\hat{c}$ = 0.83).

### Coyote use and frequency of use of prairie dog colonies

**Fig. 2.**
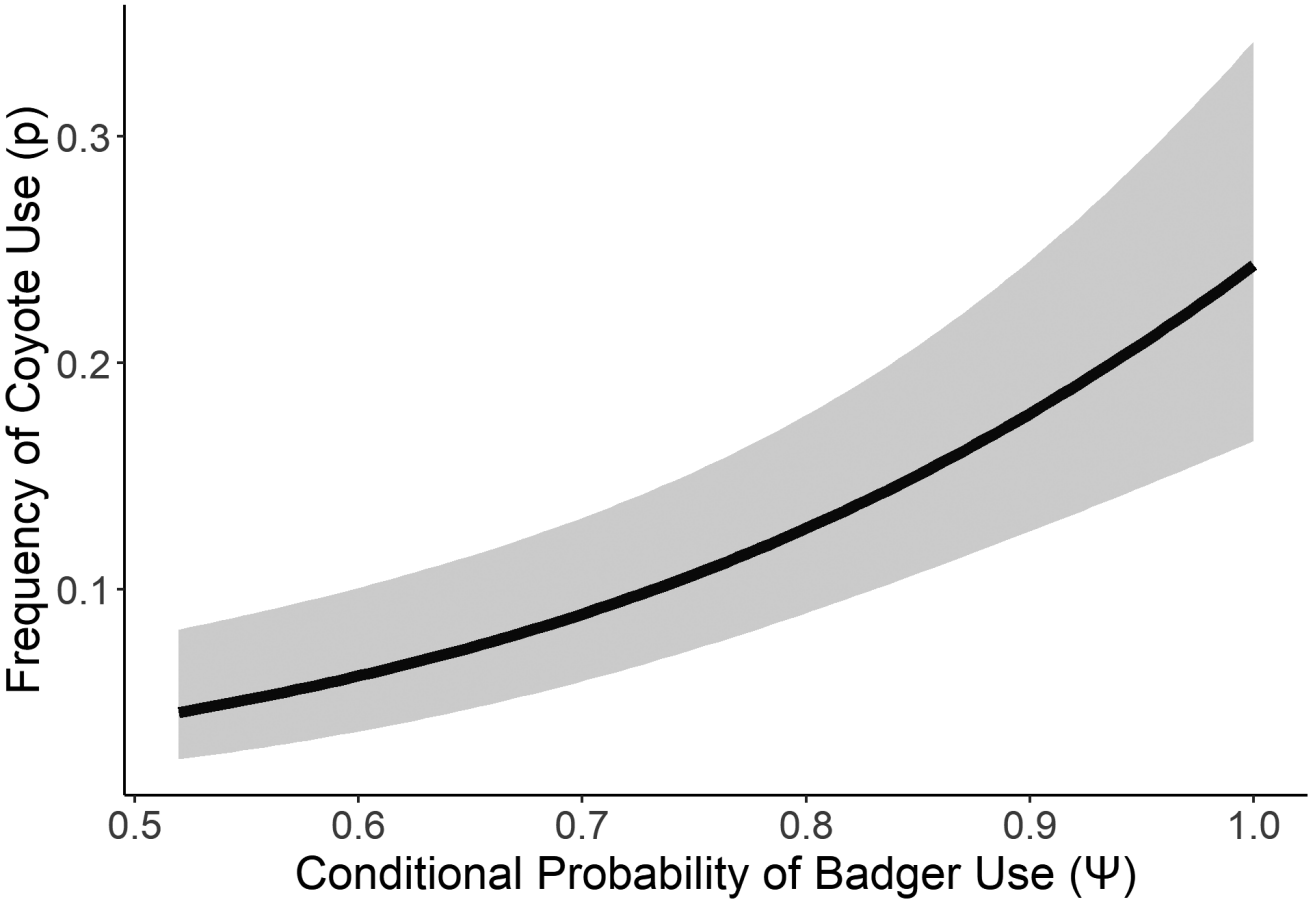
Estimated frequency of Coyote use (i.e., detection probability) was positively related to the conditional probability of badger use at camera units on prairie dog colonies in Badlands National Park and Buffalo Gap National Grassland, South Dakota in 2018.

We found little spatial variation in Coyote use among units on prairie dog colonies in our first analysis, and this was consistent in the second analysis (Table 3). Our top model suggested that Coyote use was high overall throughout prairie dog colonies with comparable estimates to those from the first analysis ($\hat{\Psi }$ = 0.89, 95% CI [0.47, 0.99]). We found that conditional badger occurrence (*Badger*) was the most important predictor of the frequency of Coyote use (Table 3); coyotes used camera units more frequently when badgers also used a unit over the course of the 30-day season ($\hat{\beta \;}$ = 3.95, 95% CI [2.35, 5.58]; Table 3; Fig. 2). Our goodness-of-fit test using the most parameterized model structure, which included badger occurrence, coarse- and fine-scale prairie dog densities, and the effect of Browning cameras ( Ψ (Badger * Coarse), *p*(Brown + Badger * Fine)), indicated good fit and no evidence of overdispersion or lack of independence amongst units ($\hat{c}$ = 0.80; χ^2^ = 1746; *P* = 0.45).

### Circadian activity

**Fig. 3.**
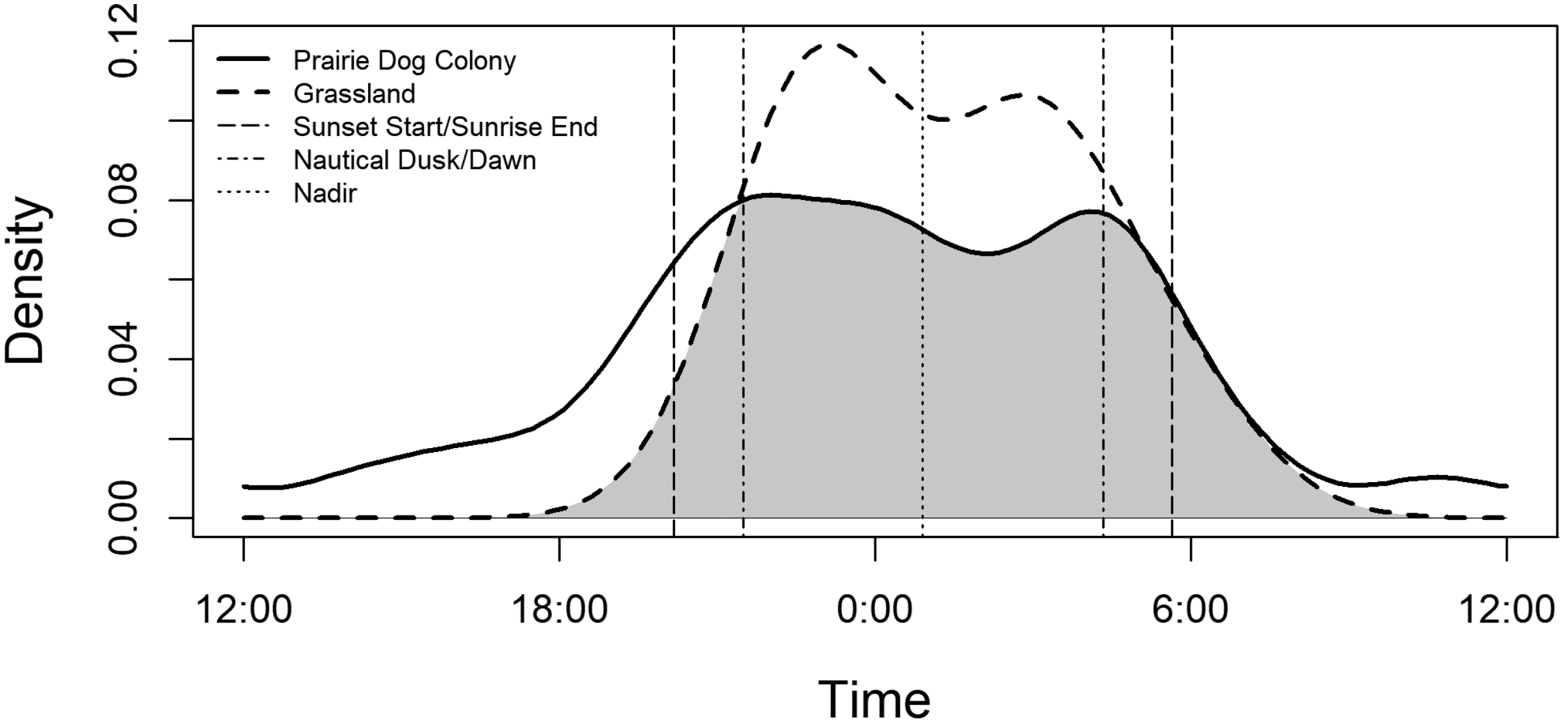
Coyote circadian activity patterns varied between cameras on prairie dog colonies (*n* = 66) and surrounding grasslands (*n* = 22) between 24 June and 30 August 2018 in Badlands National Park and Buffalo Gap National Grassland, South Dakota. Coyotes were more active on prairie dog colonies (thick solid line) than grasslands (thick dash line) during crepuscular periods of dusk (sunset start, vertical dash line to nautical dusk, vertical dot-dash line) and dawn (nautical dawn, vertical dot-dash line to sunrise start, vertical dash line), and during the day (nautical dawn to dusk). In contrast, coyotes were more active on surrounding grasslands than prairie dog colonies during both early (nautical dusk to nadir, vertical dot line) and late (nadir to nautical dawn) night hours. Note that densitiy of activity is centered on midnight (0:00).

**Fig. 4.**
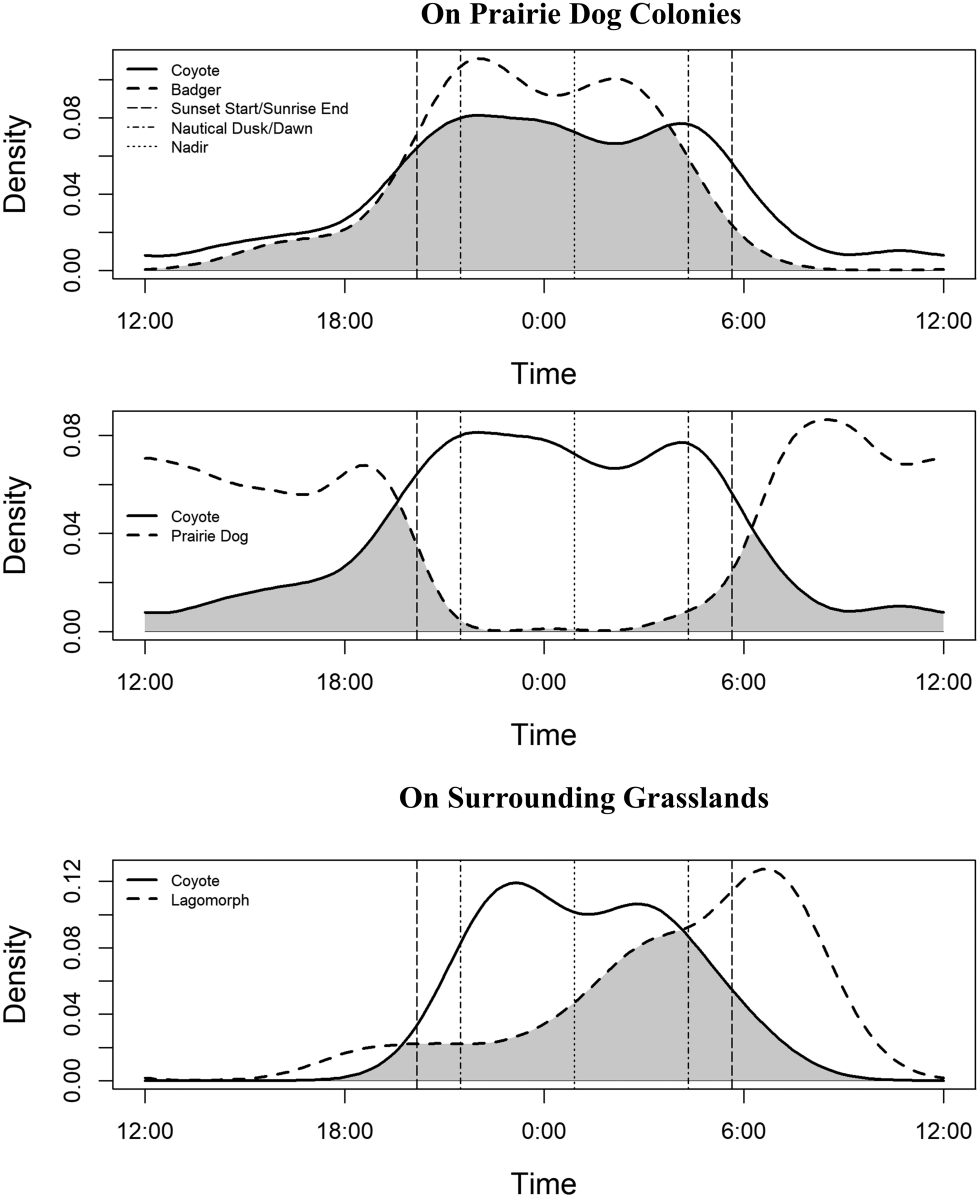
Coyote and badger activity overlap on prairie dog colonies was high (overlap = 0.81, 95% CI [0.67, 0.96]) between 24 June and 30 August 2018 in Badlands National Park and Buffalo Gap National Grassland, South Dakota. Coyotes (thick solid line) and badgers (thick dash line) were most active during both early (nautical dusk, vertical dash line, to nadir, vertical dot line) and late (nadir to nautical dawn, vertical dash line) night hours. On prairie dog colonies coyotes had low overlap with prairie dog activity patterns (overlap = 0.33, 95% CI [0.24, 0.42]). Coyotes and prairie dogs exhibited a converse pattern of onset and cessation of activity during crepuscular periods (between sunset start/sunrise end, vertical dot-dash lines, and nautical dusk/dawn) and the early part of the day (between sunrise end and sunset start). On surrounding grasslands coyotes and lagomorphs had moderate overlap in activity (overlap = 0.57, 95% CI [0.36, 0.79]) providing some evidence that coyotes may shift to surrounding grasslands to hunt lagomorphs at night. Note that densitiy of activity is centered on midnight (0:00).

Coyote circadian activity patterns varied between prairie dog colonies and surrounding grasslands (Fig. 3). In comparison to Coyote activity on grasslands, coyotes on prairie dog colonies were 1.58 and 3.32 times more active during crepuscular periods of dawn and dusk, respectively. Coyotes were also 5.65 times more likely to be active on colonies than grasslands during the day. However, coyotes were similarly active on both colonies and grasslands during both the Late Night period when ferrets are most active (0.47 times more active on colonies), and the Early Night period (0.52 times more active on colonies; Fig. 3). Coyote and badger activity overlap on prairie dog colonies was high (overlap = 0.81, 95% CI [0.67, 0.96]), and both species were most active during nocturnal periods (Fig. 4). Coyotes had low overlap with prairie dog activity patterns (overlap = 0.33, 95% CI [0.24, 0.42]), and primary overlap occurred during crepuscular periods (Fig. 4).

## Discussion

We found that coyotes with access to prairie dog colonies used these areas more than twice as much as surrounding grasslands, supporting the notion that prairie dog colonies are attractive to coyotes and used preferentially (Biggins 2000; Eads et al. 2015). The buffer of surrounding grassland around prairie dog colonies (850 m) allowed us to draw this conclusion for coyotes when their core range included colonies; however, we note that this buffer would only partially capture the core range of a Coyote that had marginal access to colonies. As a result, this limits the extent of our inference to resident coyotes with ranges that incorporate colonies or transient individuals without established ranges.

Coyotes used most camera units on prairie dog colonies ($\hat{\Psi }$ = 0.77); thus, identification of a single predictor of use (i.e., occupancy) was difficult to assess. However, over a shorter 3-day period (i.e., survey), we found that the frequency of Coyote use (i.e., detection probability) was higher at units also used by badgers (Fig. 2). This result indicates that coyotes are attracted to the presence of badgers, badger sign (e.g., excavated prairie dog burrows, which are visually conspicuous; Eldridge 2004); and/or the same resources as badgers. Furthermore, badgers and coyotes both exhibited strong overlap in diel activity patterns on colonies, with peak activity occurring during nocturnal periods for both species (Fig. 3). This result suggests that the activities of badgers may attract coyotes to specific areas on prairie dog colonies when ferrets are most active and create opportunity for direct Coyote–ferret interactions. As a result, badgers may play an important role in spatial and temporal patterns of Coyote use on prairie dog colonies, influencing the areas that coyotes use most frequently and increasing the potential for Coyote interactions with ferrets (see also Eads et al. 2015).

We hypothesized that either directly or indirectly, high prairie dog densities could attract coyotes and increase Coyote use on colonies, but we could not confidently support this hypothesis due to the high overall estimates of Coyote use on prairie dog colonies. In addition, fine-scale and coarse-scale prairie dog density were not correlated and it is possible that our results may reflect a lack of appropriate scale to assess prairie dog density as related to Coyote preferences on colonies. However, it is important to note that badger use of the landscape is likely influenced by fine-scale prairie dog densities (Eads et al. 2013, 2016). We also investigated the influence of landscape features on Coyote use of prairie dog colonies and our results support the work of Poessel et al. (2011), suggesting that neither linear landscape features nor features that provide additional cover influence how coyotes use colonies.

We found strong support that at a fine temporal scale (i.e., 3-day survey period), the frequency that coyotes used particular areas within prairie dog colonies increased as badger use increased (Fig. 2; Table 3). Our results are similar to Minta et al. (1992) and Lehner (1981) that observed badgers and coyotes hunting in mutually beneficial association, and Eads et al. (2013, 2016) who found that coyotes were attracted to the excavation activities of badgers. Badgers and ferrets both select for high-density areas of prairie dog colonies, where ferrets place strong exploitative competition pressures on badgers and badgers place strong interference competition pressures on ferrets (Eads et al. 2016). In addition, badgers selectively excavate burrows in areas used by ferrets, and ferrets appear to cache dead prairie dogs in multiple locations belowground in part as a strategy to divert the attention of kleptoparasitizing badgers (Biggins 2012; Eads et al. 2013, 2016). Although correlative, badger attraction to units used by ferrets (Eads et al. 2013) and Coyote attraction to units used by badgers may increase ferret predation risk and result in badger-mediated intraguild predation of ferrets by coyotes. However, it is important to note that we were not able to detect enough ferrets on camera to directly investigate Coyote–ferret interactions and it is thus possible that high prairie dog densities or the presence of ferrets was attracting both coyotes and badgers to these areas.

In addition to intraguild attractions, the role of lagomorphs in shaping patterns of Coyote activity and use on prairie dog colonies has been thought to have a strong influence on Coyote–ferret interactions (Biggins 2000; Eads et al. 2015). This influence is due to the importance of lagomorphs in Coyote diets and documented similarities in the crepuscular and nocturnal activity peaks of coyotes and lagomorphs (Arias-Del Razo et al. 2011). Furthermore, the cyclical nature of lagomorph populations has been hypothesized to influence the risk of ferret predation, where hazard to ferrets should decrease in periods of low lagomorph numbers as coyotes become more diurnal and shift their focus to hunting prairie dogs (Biggins 2000). Our study coincided with either a low period in the lagomorph population cycle or a significant population decline after widespread drought in the region in 2017, and we detected only a single lagomorph on prairie dog colonies during our camera trapping efforts.

We observed a relatively low sample of 19 independent detections of lagomorphs at cameras on the surrounding grasslands, where Coyote and lagomorph activity overlapped moderately (overlap = 0.57, 95% CI [0.36, 0.79]; Fig. 4). This result may help to explain why we observed higher nocturnal Coyote activity on grasslands in comparison to prairie dog colonies (Fig. 3). However, overall Coyote use of colonies was high and occurred predominantly during nocturnal periods, despite low occurrence of lagomorphs. This result suggests that during our study, Coyote use of prairie dog colonies was not a result of attraction to lagomorphs, nor did lagomorph activity on grasslands—which peaked at dawn—appear to be a strong contributor to Coyote activity patterns. Coyotes are highly adaptive and may shift their attention to lagomorphs during high periods in the population cycle when this prey source is abundant (e.g., Mech et al. 1966; Hansen and Gold 1977; Arias-Del Razo et al. 2011; Eads et al. 2015). To this end, we observed higher diurnal use of colonies by coyotes as compared to surrounding grasslands which may reflect a shift to focus on prairie dogs during low lagomorph abundance (Fig. 3). Differing trends may be expected when lagomorphs are more abundant on prairie dog colonies (Eads et al. 2015) and it is important to note that our study was unable to test this hypothesis given the limited number of lagomorph detections.

We were also interested in investigating patterns of activity between coyotes, prairie dogs, and badgers, as well as variation in diel patterns of Coyote activity on colonies in comparison to available grasslands and in relationship to the activity of ferrets. Coyotes are widely considered to be active primarily during crepuscular and nocturnal periods (e.g., Andelt and Gipson 1979; Holzman et al. 1992; Arias-Del Razo et al. 2011) and our results support this. In contrast, ferrets have strongly ingrained activity patterns, resting belowground during the daytime and becoming most active between 0100 and 0300. Biggins (2000) hypothesized that ferrets developed this behavior to reduce conflict with coyotes; however, Coyote behavior is highly plastic and activity patterns can be readily adapted (Kitchen et al. 2000). In our study area coyotes were active on prairie dog colonies during both nocturnal and crepuscular times and demonstrated flexibility in diel activity patterns.

Coyotes were 2.75 times more active on prairie dog colonies during crepuscular periods of dawn and dusk than would be expected and used prairie dog colonies during the daytime 5.65 times more than surrounding grasslands. This result suggests that coyotes are attracted to and hunt prairie dogs during their active periods (Fig. 4). Although overlap between Coyote and prairie dog activity patterns was low (i.e., 33% overlap), cessation and onset of peak Coyote activity during dawn and dusk, respectively, similarly coincided with a converse pattern of onset and cessation of prairie dog activity. Increased activity during dawn hours when prairie dogs are first emerging may be a particularly fruitful time for coyotes to hunt prairie dogs; ferrets also hunt prairie dogs emerging from burrows around dawn, which may increase their risk of predation by coyotes (Eads et al. 2010).

The bulk of Coyote activity on prairie dog colonies still occurred during both nighttime periods within the diel cycle (Fig. 3), where their activity patterns strongly overlapped those of badgers (Fig. 4) and supported the pattern that we observed in the occupancy portion of our study. However, when considering this finding it is important to note that density of badger activity was drawn from a relatively low sample of detections (*n* = 17). Coyotes were similarly active during the pre- and post-nadir nighttime periods on both prairie dog colonies and surrounding grasslands, suggesting that the canalized post-nadir activity pattern of ferrets likely provides a limited benefit to reducing interactions with coyotes.

Coyote use of prairie dog colonies, and the resulting intraguild predation of ferrets by coyotes, appears to be most strongly influenced by attraction to areas used by badgers. Coyote use of camera units was far more frequent if a unit had also been used by badgers and our study suggests that Coyote attraction to badgers may influence Coyote–ferret interactions. Further study of badger–Coyote associations and the degree to which these associations influence ferret predation risk is an important area of future research for ferret conservation, especially during reintroduction efforts.

## Supplementary data

Supplementary data are available at *Journal of Mammalogy* online.

**Supplementary Data SD1.**—We modeled the conditional probability of Badger occupancy for inclusion as a covariate in the Coyote use on prairie dog colonies versus surrounding grasslands section of this manuscript. Conditional probabilities of badger use were derived from the top model in this analysis.

**Supplementary Data SD2.**—We used a stepwise approach to investigate the influence of landscape features, prairie dog colony attributes, and interspecific attraction on Coyote use and frequency of use on prairie dog colonies. In Step 1 we modeled variables of interest on Coyote use (Ψ) and carried the top model into Step 2, where we modeled variables of interest on Coyote frequency of use (*p*). The results from this stepwise analysis were used to construct the final combined model set outlined in the Coyote use on prairie dog colonies versus surrounding grasslands section of this manuscript.

gyae066_suppl_Supplementary_Data_S1

gyae066_suppl_Supplementary_Data_S2

## Data Availability

Data files, R code, Program MARK, and Program PRESENCE files associated with this manuscript can be found at: 10.6084/m9.figshare.25852753.
