# Supplementary material for: Coyote use of prairie dog colonies is most frequent in areas used by American badgers
Source: J Mammal. 2024 Jun 28;105(6):1309–21. doi: 10.1093/jmammal/gyae066 (PMC11586099; doi:10.1093/jmammal/gyae066)
Supplement: gyae066_suppl_Supplementary_Data_S1 [file gyae066_suppl_supplementary_data_s1.docx]

Supplementary Data S1. – Badger conditional occupancy analysis

**Methods and Materials**

Convergence issues associated with limited badger detections at our camera locations precluded the use of two-species occupancy approaches to investigate our hypotheses about coyote-badger interactions (MacKenzie et al., 2017; Richmond et al., 2010). Instead, we estimated unit-specific conditional badger occupancy (i.e., the probability that badgers used the unit, given the survey results; MacKenzie et al. 2017) and used these estimates as a covariate to evaluate the effect of badger occurrence on coyote use and frequency of use. Other studies have taken a similar approach (e.g., Steen et al. 2014, Massara et al. 2018, Versiani et al. 2021). Specifically, we used a single-season occupancy framework to analyze badger detection-nondetection data collected during two 30-day rotations, 24 June to 24 July, 2018 and 31 July to 30 Aug, 2018 (total camera locations = 61). To evaluate the independent influence of badger use and other covariates on coyote use and frequency of use on colonies we used a null or constant model for badger occupancy and modeled detection probability as a function of nuisance variables, including camera type (*Bush, Brown*), the proportion of time a camera was functional during a 3-day survey occasion (*Effort*), vegetation height (*Veg*), and additive combinations of these factors (see *Nuisance variables;* Table 1).

**Results**

Our top model (AICc weight = 0.39; Table S1) indicated that badger detection varied as a result of the additive effects of Bushnell cameras ($\hat{\beta}$ = -2.31; SE = 1.05) and Effort ($\hat{\beta}$ = -0.08; SE = 0.04; Table S1)*.* We used this model structure to derive unit-specific, conditional badger occupancy probabilities (*Badger*) that were used as a covariate in our coyote analysis ($\hat{\Psi}$*_conditional_* range: 0.52-1.00). If badgers were detected at the camera unit, $\hat{\Psi}$*_conditional_* = 1. A bootstrap goodness-of-fit test (MacKenzie and Bailey 2004) using the most parameterized model structure ($\Psi$(.), *p*(Bush+Brown+Effort+Veg) yielded no sign of lack of fit (χ^2^ = 233; p-value = 0.82) or overdispersion, which would be anticipated if there was a lack of independence amongst detection histories at the camera units ($\hat{c}$ = 0.19).

Table S1.—Model selection statistics for badger detection probability structures fit to badger detection-nondetecton data from 61 camera locations in Badlands National Park and Buffalo Gap National Grasslands, South Dakota, USA. Conditional probabilities of badger use from the top model were used in models of coyote occupancy, hence we retained a null, or constant, model for badger use and only fit detection structures to account for the effect of nuisance variables. These included the potential influence of camera type (Browning = *Brown*; Bushnell = *Bush*), proportion of each survey that a camera was operational (*Effort*), and visual obstruction from vegetation (*Veg*). The plus sign (+) denotes an additive effect between covariates and the dot (.) denotes no covariate effect on Ψ or *p*. AIC_c_ = Akaike’s Information Criterion adjusted for small sample bias; w_i_ = AIC_c_ model weights; *K* = number of model parameters; Deviance = 2Log(Likelihood), a measure of model fit..

| **Model^a^** | **AIC_c_** | **Δ AIC_c_** | **w_i_** | ***K*** | **Deviance** |
| --- | --- | --- | --- | --- | --- |
| Ψ(.), *p*(Bush+Effort) | 147.32 | 0.00 | 0.39 | 4 | 138.59 |
| Ψ(.), *p*(Bush) | 149.40 | 2.08 | 0.14 | 3 | 142.97 |
| Ψ(.), *p*(Bush+Brown+Effort) ^a^ | 149.56 | 2.24 | 0.13 | 5 | 138.45 |
| Ψ(.), *p*(Bush+Effort+Veg) ^a^ | 149.65 | 2.33 | 0.12 | 5 | 138.54 |
| Ψ(.), *p*(Brown+Effort) | 151.44 | 4.12 | 0.05 | 4 | 142.71 |
| Ψ(.), *p*(Bush+Brown) ^a^ | 151.64 | 4.32 | 0.04 | 4 | 142.92 |
| Ψ(.), *p*(Bush+Veg) ^a^ | 151.70 | 4.37 | 0.04 | 4 | 142.97 |
| Ψ(.), *p*(Bush+Brown+Effort+Veg) ^a^ | 151.99 | 4.67 | 0.04 | 6 | 138.40 |
| Ψ(.), *p*(Effort) | 153.35 | 6.03 | 0.02 | 3 | 146.92 |
| Ψ(.), *p*(Brown+Effort+Veg) ^a^ | 153.80 | 6.48 | 0.02 | 5 | 142.68 |
| Ψ(.), *p*(Brown) | 155.01 | 7.69 | 0.01 | 3 | 148.58 |
| Ψ(.), *p*(.) | 155.51 | 8.19 | 0.01 | 2 | 151.30 |
| Ψ(.), *p*(Effort+Veg)^a^ | 155.63 | 8.31 | 0.01 | 4 | 146.90 |
| Ψ(.), *p*(Brown+Veg)^a^ | 157.27 | 9.95 | 0.00 | 4 | 148.54 |
| Ψ(.), *p*(Veg) | 157.71 | 10.39 | 0.00 | 3 | 151.28 |

^a^Model structures with uninformative or pretending variables (Arnold 2010).

**Acknowledgement**

Any use of trade, firm, or product names is for descriptive purposes only and does not imply endorsement by the U.S. Government.

**References**

Arnold TW. 2010. Uninformative parameters and model selection using Akaike's Information Criterion. *The Journal of Wildlife Management*, *74*(6): 1175-1178. https://doi.org/10.1111/j.1937-2817.2010.tb01236.x

MacKenzie DI, Bailey LL. 2004. Assessing the fit of site-occupancy models. Journal of Agricultural, Biological, and Environmental Statistics 9:300–318. https://doi.org/10.1198/108571104X3361

MacKenzie DI, Nichols JD, Royle JA, Pollock KH, Bailey LL, Hines JE. 2017. Occupancy estimation and modeling: inferring patterns and dynamics of species occurrence. Amsterdam (Netherlands): Elsevier.

Massara RL, Paschoal AMDO, Bailey LL, Doherty PF, Hirsch A, Chiarello AG. 2018. Factors influencing ocelot occupancy in Brazilian Atlantic Forest reserves. *Biotropica* *50*(1): 125-134. https://doi.org/10.1111/btp.12481

Richmond OM, Hines JE, Beissinger S. 2010. Two‐species occupancy models: a new parameterization applied to co‐occurrence of secretive rails. *Ecological applications* *20*(7): 2036-2046. https://doi.org/10.1890/09-0470.1

Steen DA, McClure CJ, Brock JC, Rudolph DC, Pierce, Lee JR, Humphries WJ, Gregory BB, Sutton WB, Smith LL, et al. 2014. Snake co‐occurrence patterns are best explained by habitat and hypothesized effects of interspecific interactions. *Journal of Animal Ecology* *83*(1): 286-295. https://doi.org/10.1111/1365-2656.12121

Versiani NF, Bailey LL, Pasqualotto N, Rodrigues TF, Paolino RM, Alberici V, Chiarello AG. 2021. Protected areas and unpaved roads mediate habitat use of the giant anteater in anthropogenic landscapes. Journal of Mammalogy 102(3): 802-813. https://doi.org/10.1093/jmammal/gyab004
