# Supplementary material for: Coyote use of prairie dog colonies is most frequent in areas used by American badgers
Source: J Mammal. 2024 Jun 28;105(6):1309–21. doi: 10.1093/jmammal/gyae066 (PMC11586099; doi:10.1093/jmammal/gyae066)
Supplement: gyae066_suppl_Supplementary_Data_S2 [file gyae066_suppl_supplementary_data_s2.docx]

Supplementary Data S2. – Stepwise model building approach to investigate factors influencing coyote use and frequency of use on prairie dog colonies.

**Step 1**: **Evaluating factors influencing coyote use**

**Methods**

We modeled coyote use (Ψ) of prairie dog colonies as a function of badger use (*Badger*), fine- (*PD Fine*) and coarse-scale *(PD Coarse*) prairie dog density, size of prairie dog colony (*Size*); distance of camera from edge of colony (*Edge*), riparian areas (*Rip*), streams (*Stream*), and two-track roads (*Two-Track*); and a null model (.). We also included additive effects (+) of prairie dog density metrics and badger use or camera distance from the edge of the colony. Finally, we included interactive effects (*) between badger use and the two prairie dog density metrics. All detection structures included the additive effect of Browning cameras (*Brown*) on frequency of coyote use (*p*) based on a concurrent study (Windell et al. 2021) and the results from our first analysis. We excluded the effect of *Rotation* on coyote use based the results on our first analysis as it resembled an uninformative, or pretending, variable (Arnold 2010).

The null model, where coyote use and frequency of use are constant across all units and surveys, Ψ(.), p(.), is given as a reference.

**Results**

We carried the top model which included the effect of *Badger* on coyote use to step two of the analysis (Supplementary Data S3); however, we retained all use structures without uninformative variables (i.e., those that had similar deviances to less parameterized models, Arnold 2010) that were better supported than the null model in the final, combined model set.

Table. S2a. – Model selection results for coyote use (Ψ) on 8 prairie dog colony subcomplexes with known black-footed ferret presence in Badlands National Park and Buffalo Gap National Grasslands, South Dakota in 2018. Model selection statistics include: AIC_c_ = Akaike’s Information Criterion adjusted for small sample bias; w_i_ = AIC_c_ model weights; *K* = number of model parameters; Deviance = 2Log(Likelihood), a measure of model fit. Structures retained in the final, combined model set are those above the horizontal line.

| **Model^a^** | **AIC_c_** | **Δ AIC_c_** | **w_i_** | ***K*** | **Deviance** |
| --- | --- | --- | --- | --- | --- |
| Ψ(Badger), *p*(Brown) | 322.97 | 0.00 | 0.30 | 4 | 314.26 |
| Ψ(Badger+PD Coarse), *p*(Brown) | 324.15 | 1.18 | 0.17 | 5 | 313.06 |
| Ψ(Badger+PD Fine), *p*(Brown)^a^ | 325.32 | 2.35 | 0.09 | 5 | 314.23 |
| Ψ(Badger*PD Coarse), *p*(Brown) | 326.03 | 3.06 | 0.07 | 6 | 312.47 |
| Ψ(PD Fine), *p*(Brown) | 326.12 | 3.15 | 0.06 | 4 | 317.41 |
| Ψ(PD Coarse), *p*(Brown) | 326.43 | 3.45 | 0.05 | 4 | 317.71 |
| Ψ(.), *p*(Brown) | 326.84 | 3.86 | 0.04 | 3 | 320.42 |
| Ψ(.), *p*(.) | 327.04 | 4.07 | 0.04 | 2 | 322.83 |
| Ψ(Badger*PD Fine), *p*(Brown) | 327.44 | 4.47 | 0.03 | 6 | 313.89 |
| Ψ(Edge+PD Fine), *p*(Brown) | 327.51 | 4.54 | 0.03 | 5 | 316.42 |
| Ψ(Edge+PD Coarse), *p*(Brown) | 328.07 | 5.10 | 0.02 | 5 | 316.98 |
| Ψ(Size), *p*(Brown) | 328.54 | 5.57 | 0.02 | 4 | 319.83 |
| Ψ(Edge), *p*(Brown) | 329.06 | 6.09 | 0.01 | 4 | 320.35 |
| Ψ(Two-Track), *p*(Brown) | 329.12 | 6.14 | 0.01 | 4 | 320.40 |
| Ψ(Rip), *p*(Brown) | 329.13 | 6.16 | 0.01 | 4 | 320.41 |
| Ψ(Stream), *p*(Brown) | 329.13 | 6.16 | 0.01 | 4 | 320.42 |

^a^Model structures with uninformative or pretending variables (Arnold 2010).

**Step 2. – Evaluating factors influencing frequency of use by coyotes at units on prairie dog colonies**

**Methods**

We used the most parsimonious occupancy (use) structure from step 1, which included the effect of *Badger* on coyote use, to evaluate factors influencing detection probability, or the frequency of coyote use (*p*). We modeled the frequency of coyote use as a function of badger use (*Badger*), fine- (*PD Fine*) and coarse-scale *(PD Coarse*) prairie dog density, size of prairie dog colony (*Size*), distance of camera from edge of colony (*Edge*), riparian areas (*Rip*), streams (*Stream*), and two-track roads (*Two-Track*); and a null model (.). We also included additive effects (+) of prairie dog density metrics and badger use or camera distance from the edge of the colony, and distance to two-track roads and riparian areas with streams,. Finally, we included interactive effects (*) between badger use and the two prairie dog density metrics, and between colony size and camera distance to the edge of the colony. All detection structures included the additive effect of Browning cameras (*Brown*) on frequency of coyote use.

**Results**

The best supported model (*w* = 0.48) suggested that coyote frequency of use was influenced by Badger occurrence, after controlling for camera type. We retained only this detection structures in the final, combined model set, as all other supported structures (*w* > 0.10) contained uninformative variables (Arnold 2010).

Table. S2b. – Model selection results for frequency of coyote use (*p*) on 8 prairie dog colony subcomplexes with known black-footed ferret presence in Badlands National Park and Buffalo Gap National Grasslands, South Dakota in 2018 using a stepwise approach.. Model selection statistics include: AIC_c_ = Akaike’s Information Criterion adjusted for small sample bias; w_i_ = AIC_c_ model weights; *K* = number of model parameters; Deviance = the difference between the proposed and saturated models.

| **Model^a^** | **AIC_c_** | **Δ AIC_c_** | **w_i_** | ***K*** | **Deviance** |
| --- | --- | --- | --- | --- | --- |
| Ψ(Badger), *p*(Brown+Badger) | 311.38 | 0.00 | 0.48 | 5 | 300.29 |
| Ψ(Badger), *p*(Brown+Badger+Fine) ^a^ | 313.59 | 2.22 | 0.16 | 6 | 300.04 |
| Ψ(Badger), *p*(Brown+Badger+Coarse) ^a^ | 313.69 | 2.32 | 0.15 | 6 | 300.14 |
| Ψ(Badger), *p*(Brown+Badger*Fine) | 314.80 | 3.43 | 0.09 | 7 | 298.69 |
| Ψ(Badger), *p*(Brown+Badger*Coarse)^a^ | 316.23 | 4.86 | 0.04 | 7 | 300.12 |
| Ψ(Badger), *p*(Brown+Rip) | 316.54 | 5.16 | 0.04 | 5 | 305.45 |
| Ψ(Badger), *p*(Brown+Fine) | 318.39 | 7.01 | 0.01 | 5 | 307.30 |
| Ψ(Badger), *p*(Brown+2Track) | 318.56 | 7.18 | 0.01 | 5 | 307.46 |
| Ψ(Badger), *p*(Brown+Rip+Stream) | 319.00 | 7.62 | 0.01 | 6 | 305.44 |
| Ψ(Badger), *p*(Brown+Edge+Fine) | 320.51 | 9.13 | 0.00 | 6 | 306.95 |
| Ψ(Badger), *p*(Brown+2Track+Stream) | 320.83 | 9.45 | 0.00 | 6 | 307.27 |
| Ψ(Badger), *p*(Brown+Edge) | 322.47 | 11.10 | 0.00 | 5 | 311.38 |
| Ψ(Badger), *p*(Brown) | 322.97 | 11.60 | 0.00 | 4 | 314.26 |
| Ψ(Badger), *p*(Brown+Stream) | 324.25 | 12.87 | 0.00 | 5 | 313.16 |
| Ψ(Badger), *p*(Brown+Edge+Coarse) | 324.93 | 13.55 | 0.00 | 6 | 311.37 |
| Ψ(Badger), *p*(Brown+Size) | 325.00 | 13.63 | 0.00 | 5 | 313.91 |
| Ψ(Badger), *p*(Brown+Badger 1-10) | 325.11 | 13.74 | 0.00 | 5 | 314.02 |
| Ψ(Badger), *p*(Brown+Coarse) | 325.14 | 13.76 | 0.00 | 5 | 314.05 |
| Ψ(Badger), *p*(Brown+Size*Edge) | 326.83 | 15.46 | 0.00 | 7 | 310.72 |

^a^Model structures with uninformative or pretending variables (Arnold 2010).

**Acknowledgement**

Any use of trade, firm, or product names is for descriptive purposes only and does not imply endorsement by the U.S. Government.

**References**

Arnold TW. 2010. Uninformative parameters and model selection using Akaike's Information Criterion. *The Journal of Wildlife Management*, *74*(6): 1175-1178. https://doi.org/10.1111/j.1937-2817.2010.tb01236.x
